# Pyrosequencing Characterization of the Microbiota from Atlantic Intertidal Marine Sponges Reveals High Microbial Diversity and the Lack of Co-Occurrence Patterns

**DOI:** 10.1371/journal.pone.0127455

**Published:** 2015-05-20

**Authors:** Anoop Alex, Agostinho Antunes

**Affiliations:** 1 CIIMAR/CIMAR, Interdisciplinary Centre of Marine and Environmental Research, University of Porto, Rua dos Bragas 177, 4050–123, Porto, Portugal; 2 Department of Biology, Faculty of Sciences, University of Porto, Rua do Campo, Alegre, 4169–007, Porto, Portugal; Laval University, CANADA

## Abstract

Sponges are ancient metazoans that host diverse and complex microbial communities. Sponge-associated microbial diversity has been studied from wide oceans across the globe, particularly in subtidal regions, but the microbial communities from intertidal sponges have remained mostly unexplored. Here we used pyrosequencing to characterize the microbial communities in 12 different co-occurring intertidal marine sponge species sampled from the Atlantic coast, revealing a total of 686 operational taxonomic units (OTUs) at 97% sequence similarity. Taxonomic assignment of 16S ribosomal RNA tag sequences estimated altogether 26 microbial groups, represented by bacterial (75.5%) and archaeal (22%) domains. *Proteobacteria* (43.4%) and *Crenarchaeota* (20.6%) were the most dominant microbial groups detected in all the 12 marine sponge species and ambient seawater. The *Crenarchaeota* microbes detected in three Atlantic Ocean sponges had a close similarity with *Crenarchaeota* from geographically separated subtidal Red Sea sponges. Our study showed that most of the microbial communities observed in sponges (73%) were also found in the surrounding ambient seawater suggesting possible environmental acquisition and/or horizontal transfer of microbes. Beyond the microbial diversity and community structure assessments (NMDS, ADONIS, ANOSIM), we explored the interactions between the microbial communities coexisting in sponges using the checkerboard score (C-score). Analyses of the microbial association pattern (co-occurrence) among intertidal sympatric sponges revealed the random association of microbes, favoring the hypothesis that the sponge-inhabiting microbes are recruited from the habitat mostly by chance or influenced by environmental factors to benefit the hosts.

## Introduction

Sponge microbial ecology is attaining momentum with the advancement of sequencing technology and the keen interest in unlocking the global sponge-associated microbial diversity. The sponge-associated microorganisms can contribute to nearly 40% of the total sponge biomass [1] and may benefit the hosts with various functional roles. Culture-independent 16S rRNA (ribosomal RNA) gene based molecular techniques, such as Sanger sequence analyses derived from clone library [2–4], denaturing gradient gel electrophoresis [5], terminal restriction fragment length polymorphism [3,6] and culture-dependent isolation procedures [2,4] provided insight into the complex sponge microbial consortium. In recent years, 454 tag sequencing of the 16S rRNA revealed an unexplored exceptional diversity of microbial assemblages residing within the sponge body [7–9]. Apart from cataloging the microbial diversity, the potential pharmacological application of bioactive compounds isolated from the sponges and associated microbes further intensified the effort to understand the sponge-associated microbial world [10].

Irrespective of the enormous amount of microbial community data from widely distributed sponges, the mechanism of symbiotic establishment and its exact role within the sponge host remains mostly unclear [11,12]. It has been suggested that microbial interaction may provide the sponges with benefits that include the incorporation of dissolved organic matter [13,14], production of photosynthates [15,16], antifeedants [17–20] and removal of waste products [21–23]. Sponge-associated microbes has been classified as photosynthetic bacteria [24], heterotrophic bacteria, archaea [25] and non-representative candidate phyla [26,27].

Sponge-microbe association, its variability and similarity have been assessed by the sampling of (i) distinct sponge species from different oceans [9], (ii) congeneric sponges from different oceans [28] (iii) congeneric sponges from the same ocean but sampled from neighboring sites [29] and (iv) sympatric distantly related sponges from the same habitat [30]. Early studies hypothesized that sponges host a “uniform bacterial community” [31,32], however subsequent studies questioned the true nature of these microbes due to the presence of sponge-specific microbes in seawater samples [7]. Further investigation of sponge-microbe association detected the existence of common symbiotic microbial taxa across various sponge lineages, suggesting the lack of host specificity and described a ‘mix of specialist and generalist’ microbiota among sponge hosts [3,4,29]. However, beyond alpha and beta diversity analyses, to our knowledge no studies have been performed to examine the interactions between microbial taxa (co-occurrence pattern) coexisting in sponge hosts. Determining the bacterial community structure and structure-function relationship is crucial to infer the ecological niche of bacterial taxa and their interactions [33–35].

Here, we have used 454 pyrosequencing (i) to characterize the bacterial community from 12 different intertidal marine sponge species (class Demospongiae) inhabiting the same habitat (single sampling site collection) in the Atlantic coast of Portugal and (ii) to determine co-occurrence patterns among the microbial communities in sponges. In depth sequencing from unexplored Atlantic intertidal marine sponges revealed the presence of diverse microbial consortium including several groups of bacteria, candidate phyla and *Crenarchaeota* group. Crenarchaeotal symbionts associated with sponge species in our current study were compared with to those associated with sponges in the Red Sea to test for symbiont similarity between geographically and taxonomically distinct sponges. Furthermore, we tested for the nature of the association (random or non-random) prevailing among the microbial communities in sponge species.

## Materials and Methods

### Ethics statement

The study did not involve any kind of endangered or protected species. No specific scientific research permits were required to conduct the field study and sampling of the sponges from the rocky beaches.

### Sample collection and 454 pyrosequencing

Sampling of different sponge species (while sponges were exposed during low tide, n = 12, Table 1) and surrounding ambient seawater (one or two specimens each) has been performed on 14^th^ January 2013 at Praia da Memória (41.2308206N 8.7216926W), an Atlantic Ocean rocky beach from Portugal. Within one hour of sampling and transportation to the lab in an insulated container, sponge tissues of 1cm^3^ were washed thoroughly with sterile seawater prior to DNA extraction. Approximately 2.5 liters of seawater from the sample location was filtered through 0.45μm sterile filter followed by DNA extraction with PureLink^TM^ Genomic DNA kit (Invitrogen). Amplicon libraries for 454 pyrosequencing were constructed with unique barcoded (S1 Table) universal primers U789F (5'-TAGATACCCSSGTAGTCC-3') and reverse primer U1068R (5'-CTGACGR CRGCCATGC-3') 16S rRNA gene (Baker et al. 2003) targeting at hypervariable region V6 of bacteria and archaea. Triplicate PCR reactions were performed in a total volume of 100 μl constituting 5 U of *Pfx*50 DNA polymerase (Invitrogen), 1X *Pfx*50 PCR mix, 0.3 mM of dNTPs (NZYTech), 0.5 μM of each barcoded primers and 30 ng of metagenomic DNA. Thermocycler conditions, initial denaturation at 95°C for 5 minutes and 26 cycles of 94°C for 15 seconds, 63°C for 30 seconds, 68°C for 45 seconds and a final extension at 68°C for 5 minutes were used. Gel purified PCR (Macherey-Nagel) products were pooled and performed pyrosequencing on ROCHE 454 GS-FLX Titanium platform. Raw pyrosequencing reads were submitted to the NCBI Short Reads Archive database (SRR949132).

**Table 1:** List of sponge species collected.

			Taxonomy	
Sponge species	Sample code	Class	Order	Family
*Amphilectus fucorum*	AMF (n = 1)	Demospongiae	Poecilosclerida	Esperiopsidae
*Aplysilla rosea*	APL (n = 1)	Demospongiae	Dendroceratida	Darwinellidae
*Aaptos papillata*	AAP (n = 2)	Demospongiae	Hadromerida	Suberitidae
*Cliona celata*	CCL (n = 1)	Demospongiae	Hadromerida	Clionaidae
*Haliclona simulans*	HAS (n = 2)	Demospongiae	Haplosclerida	Chalinidae
*Halichondria panicea*	HAL (n = 2)	Demospongiae	Halichondrida	Halichondriidae
*Ophlitaspongia papilla*	OPT (n = 2)	Demospongiae	Poecilosclerida	Microcionidae
*Polymastia agglutinans*	PAG (n = 2)	Demospongiae	Hadromerida	Polymastiidae
*Polymastia penicillus*	POLY (n = 2)	Demospongiae	Hadromerida	Polymastiidae
*Polymastia* sp.	POL (n = 1)	Demospongiae	Hadromerida	Polymastiidae
*Phorbas plumosus*	PHR (n = 2)	Demospongiae	Poecilosclerida	Hymedesmiidae
*Tedania pillarriosae*	TED (n = 2)	Demospongiae	Poecilosclerida	Tedaniidae

Intertidal marine sponge species collected from the Portuguese Atlantic coast and their respective sample codes. Number in parentheses represents number of specimens used for the study. Ambient seawater collected from the same sampling location was labeled with the code SW.

### 454 tag sequence processing and OTU picking

Pyrosequencing data analyses were performed with QIIME v.1.6.0 [36]. Briefly, raw multiplexed sequences (138,615 reads) were pre-processed by trimming with an average quality threshold score of 25, removing reads containing ambiguous bases, sequences shorter than 100 bp and unassigned reads. Final sequences of an average read length of 285.5 bp were assigned to samples based on barcodes for downstream analysis. Before proceeding with diversity analysis, pre-processed dataset was screened by denoising [37] to avoid over representation of species diversity. De-multiplexed reads were checked for chimeras using UCHIME [38] against 16S “Gold” database (reference database in the Broad Microbiome Utilities, version microbiomeutil-r20110519; http://microbiomeutil.sourceforge.net/) and clustered into operational taxonomic units (OTUs) using a 97% similarity threshold with USEARCH algorithm [39]. Taxonomic assignment to phylum, class, order, family and genus level was implemented with naïve Bayesian classifier [40] on a set of trained Greengenes reference sequences and taxonomy [41] by mothur method at 80% similarity confidence [42].

### Microbial diversity and co-occurrence analysis

The BIOM (Biological Observation Matrix) file obtained after clustering the reads at 97% similarity was further used for downstream analysis. Briefly, microbial richness indices namely observed species richness (S.obs), expected richness with Chao1 estimator (S.Chao1) [43], abundance-based coverage estimator (S.ACE) [44] and diversity measures (shannon and simpson indices) [45,46] were executed by plot_richness function in phyloseq package v 1.5.15 [47]. The non-metric multidimensional scaling (NMDS) was performed to visually compare the microbial community dissimilarity among different sponge species and the ambient seawater using Bray-Curtis distance.

Non-random association pattern of microbes among sponges was tested by calculating the checkerboard score (C-score) [48] using the co-occurrence [49] module in EcoSim v 7.71 [50]. We evaluated this relationship between microbes that were identified as core i.e., OTUs present in at least 50% of the sponge samples. Data was transformed into a presence-absence matrix (PAM) and was used to generate the C-score [48] under a null model. Representation using PAM could be insightful and useful, particularly when the association patterns shaping the species distribution are influenced mostly by both environmental and ecological factors [51]. Observed C-scores were compared with expected C-scores by fixed rows-equiprobable columns simulation algorithm. We used the C-score to investigate whether the microbes are randomly distributed among the sponge samples. Significantly larger observed C-score than the expected C-score indicates the possible segregation (non-random pattern) of microbial taxa and smaller observed C-score than the expected C-score implies the possible aggregation (random pattern) of microbes.

### Crenarchaeota microbial community from the Atlantic Ocean and the Red Sea sponges

Due to the high abundance of *Crenarchaeota* in our study, we performed a comparative case study to evaluate the similarity of *Crenarchaeota* from our study and that previously detected in the Red Sea sponges [8]. Same read length size of 16S rRNA amplicons (~ 300 bp encompassing the V6 hypervariable region) from the Atlantic Ocean (current samples) and the Red Sea samples, improved the taxonomic resolution and diversity analyses. We retrieved the raw pyrosequencing data of the Red Sea sponges (SRA012874.2) from the NCBI Short Reads Archive database. The sequencing reads were pre-processed with NCBI SRA toolkit (http://www.ncbi.nlm.nih.gov/Traces/sra/sra.cgi?view=software) and analyzed as mentioned above using QIIME v.1.6.0 [36].

Reads assigned to *Crenarchaeota* were retrieved from both data sets and merged to generate a final OTU table for further analyses. We employed UPGMA (Unweighted Pair Group Method using arithmetic Averages) cluster analysis [52] with the Crenarchaeotal dataset using Bray-Curtis distance matrix. UPGMA performed the clustering of the microbes (*Crenarchaeota*) represented in different samples based on the overall similarities among the microbial taxa. The closely related sponge species are grouped together and illustrated as a dendrogram. Statistical significance of the sample groupings (Atlantic Ocean and Red Sea samples) was tested with nonparametric ADONIS and ANOSIM functions using the vegan package v 2.0.7 [53] in QIIME v.1.6.0 [36]. We used ADONIS, a robust permutation analyses to test the differentiation between the means of two or more groups of data, to explain the percentage of variation by computing the effective size (*R* ^*2*^) and a *p*-value. Whereas, the ANOSIM tests whether two groups (Atlantic Ocean and Red Sea samples) are significantly different by comparing the ranks of distances between the groups and within the groups. Analyses were conducted with 1000 permutations.

## Results

### OTUs and bacterial richness

**Fig 1:**
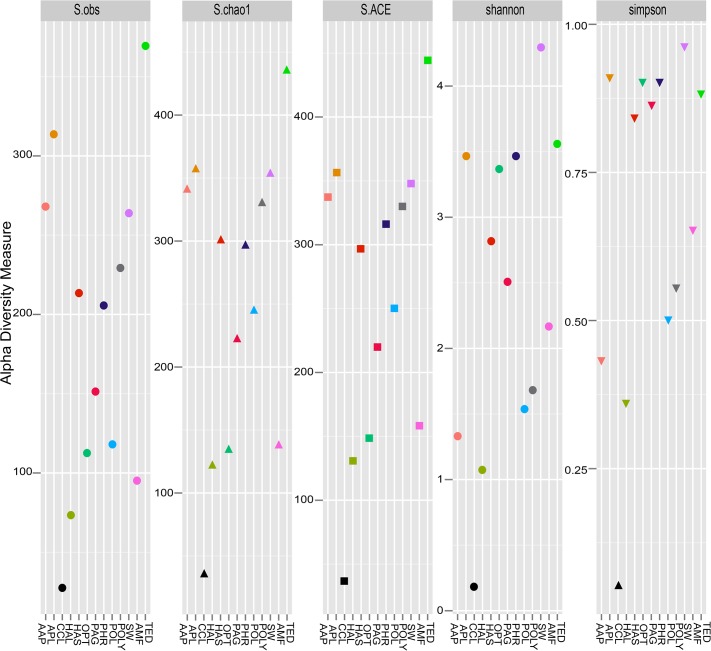
Alpha diversity indices of microbial communities from the 12 sponge species and seawater (SW). Community richness was estimated using observed species (S.obs), chao 1 estimator (S.chao1) and abundance-based coverage estimator (S.ACE). Community diversity was calculated using Shannon and Simpson indices. Analyses were executed with phyloseq package v 1.5.15 [47] in the R environment (http://www.R-project.org/) and plotted using ggplot2 [85]. Sample description is provided in Table 1.

454 sequencing and quality sorting of the microbial communities associated with the sponges and seawater derived 84,199 sequence reads clustered at 97% identity into 686 unique OTUs. Clustering of the reads at lower sequence similarity thresholds resulted in lower number of OTUs (S1 Fig). The total number of reads retrieved and OTUs (at 3% sequence divergence) from each sample are provided in S2 Fig. The highest number of OTUs was observed in the sponge *Amphilectus fucorum* (AMF), representing up to 370 OTUs and minimum of 29 OTUs from the sponge *Cliona celata* (CCL) (Fig 1). OTU based alpha diversity measures, abundance-based coverage estimator (ACE) and Chao1 presented the higher richness of microbial species in the sponge AMF and lowest in the sponge CCL. Most of the sponge species and seawater (SW) showed richness ranging from 100–400. Microbial community diversity, Shannon and Simpson indices indicated higher dominance in seawater (SW) compared to the sponge species sampled from the same location (Fig 1). Rank-abundance curve suggested the presence of a few abundant bacterial species in the sponge samples and in the surrounding seawater (S3 Fig).

### Taxonomic composition of pyrosequencing reads

**Fig 2:**
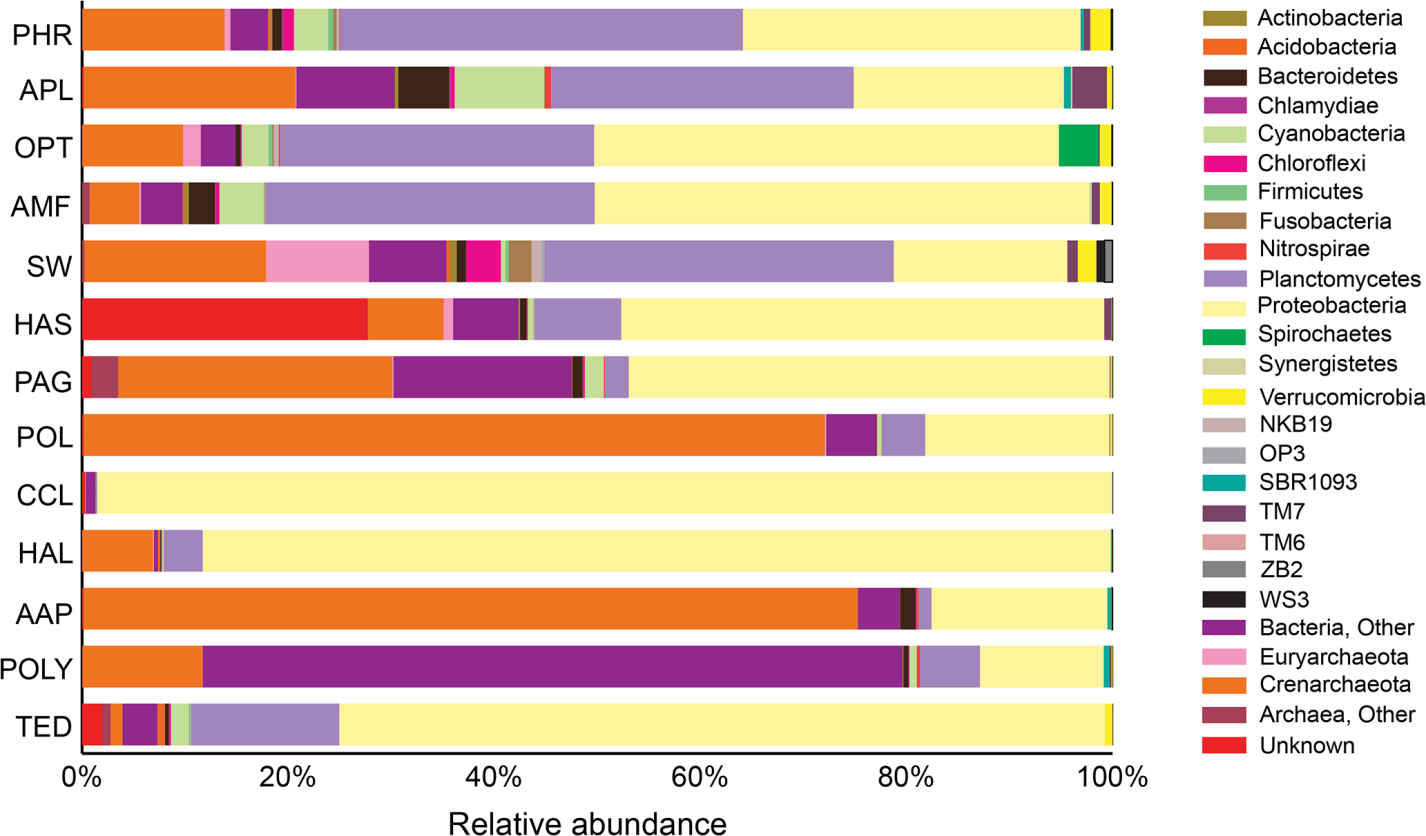
Taxonomic compositions and relative abundance of the OTUs from the sponge species and surrounding seawater. Different color pellets are assigned to represent bacterial groups. Detailed information about sample codes is provided in Table 1.

Taxonomic assignment of qualified reads (84,199) with Greengenes reference sequences at a confidence threshold of 97% similarity classified the reads into two major domains- bacteria (75.5%) and archaea (22.0%), with 22 bacterial, 3 archaeal and 1 unknown group. The known bacterial phyla- *Actinobacteria*, *Acidobacteria*, *Bacteroidetes*, *Chlamydiae*, *Cyanobacteria*, *Chloroflexi*, *Firmicutes*, *Fusobacteria*, *Nitrospirae*, *Planctomycetes*, *Proteobacteria*, *Spirochaetes*, *Synergistetes*, *Verrucomicrobia* and several candidate bacterial phyla- NKB19, OP3, SBR1093, TM7, TM6, ZB2 and WS3 were detected in the sponge samples and ambient seawater (Fig 2). The bacterial group *Proteobacteria* dominated in all samples comprising the major class- *Alphaproteobacteria* (12.1%), *Gammaproteobacteria* (10.6%), *Betaproteobacteria* (2.7%), *Deltaproteobacteria* (1.3%), other Proteobacteria (16.7%) and least represented by *Epsilonproteobacteria* (0.2%), but only in the sponge AMF (S4 Fig). *Planctomycetes* (15.8%) and *Cyanobacteria* (1.9%) were also retrieved from the sponges studied. The photosynthetic bacterial reads affiliated to *Prochlorococcus* were present in all samples except in the sponges *Aaptos papillata* (AAP) and *C*. *celata*.

In this study both seawater and sponges harbored the archaeal group of the phylum *Crenarchaeota* (20.6%) and *Euryarchaeota* (1.1%) (Fig 2, S5A Fig). *Crenarchaeota* were observed with high frequency in all samples, whereas, *Euryarchaeota* reads were found in lower abundance and were notably absent in a few sponge species including CCL, AAP, POLY and TED (S5A Fig). *Nitrosopumilus* sp. was the most abundant archaeon found among the samples analyzed in this study (S5B Fig).

### Bacterial community similarity and random distribution

**Fig 3:**
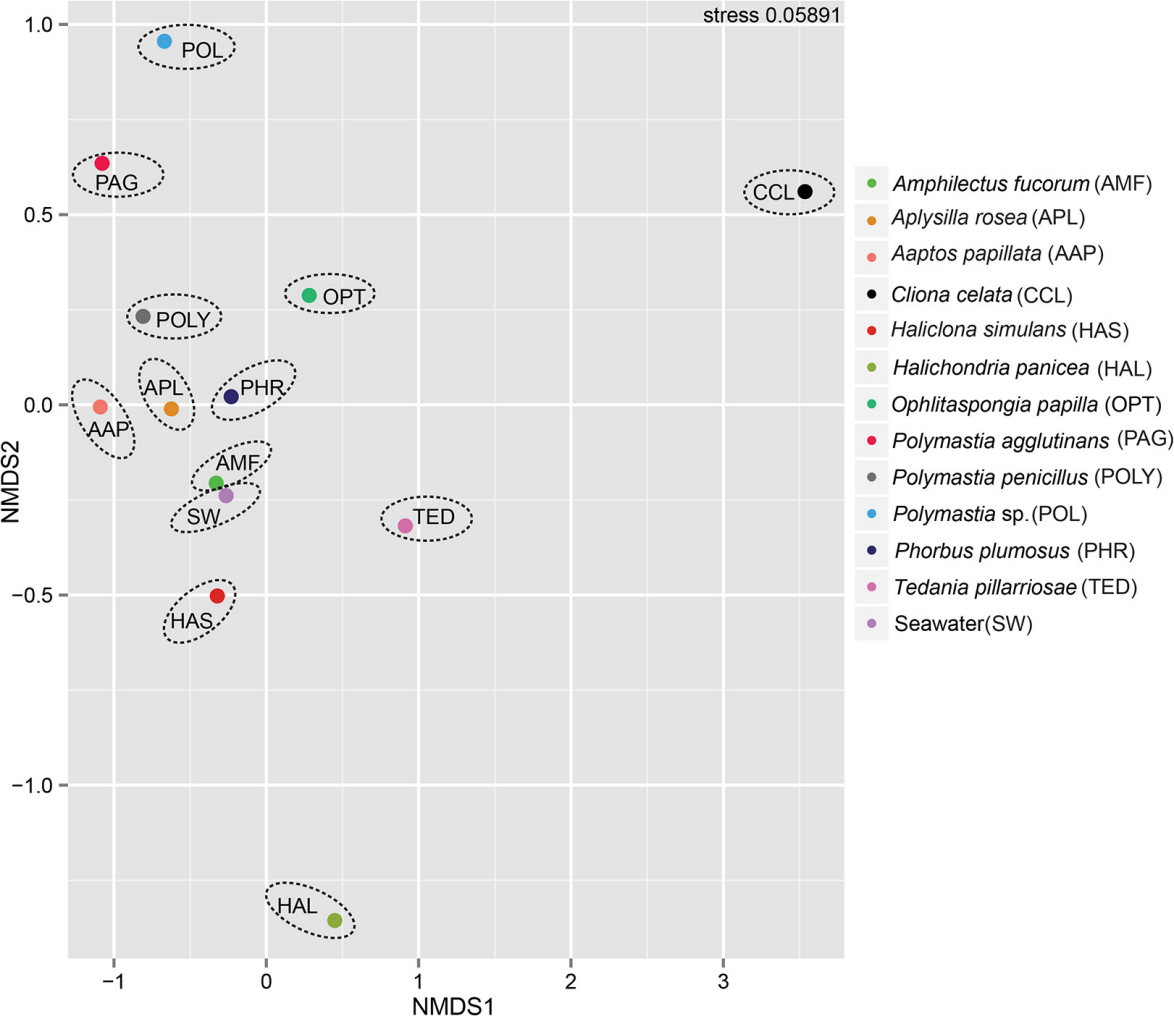
Microbial community differentiations among the intertidal Atlantic Ocean samples. Non-metric multidimensional scaling plots showing the pattern of microbial communities recovered from the sponge samples and seawater. Each sample code and its designated colored dots were delimited by an ellipse for visualization purpose. Stress value < 0.05 shows an excellent representation in NMDS analysis, while stress value < 0.01 gives a good representation. Analyses were executed with phyloseq package v 1.5.15 [47] in the R environment (http://www.R-project.org/) and plotted using ggplot2 [85].

**Fig 4:**
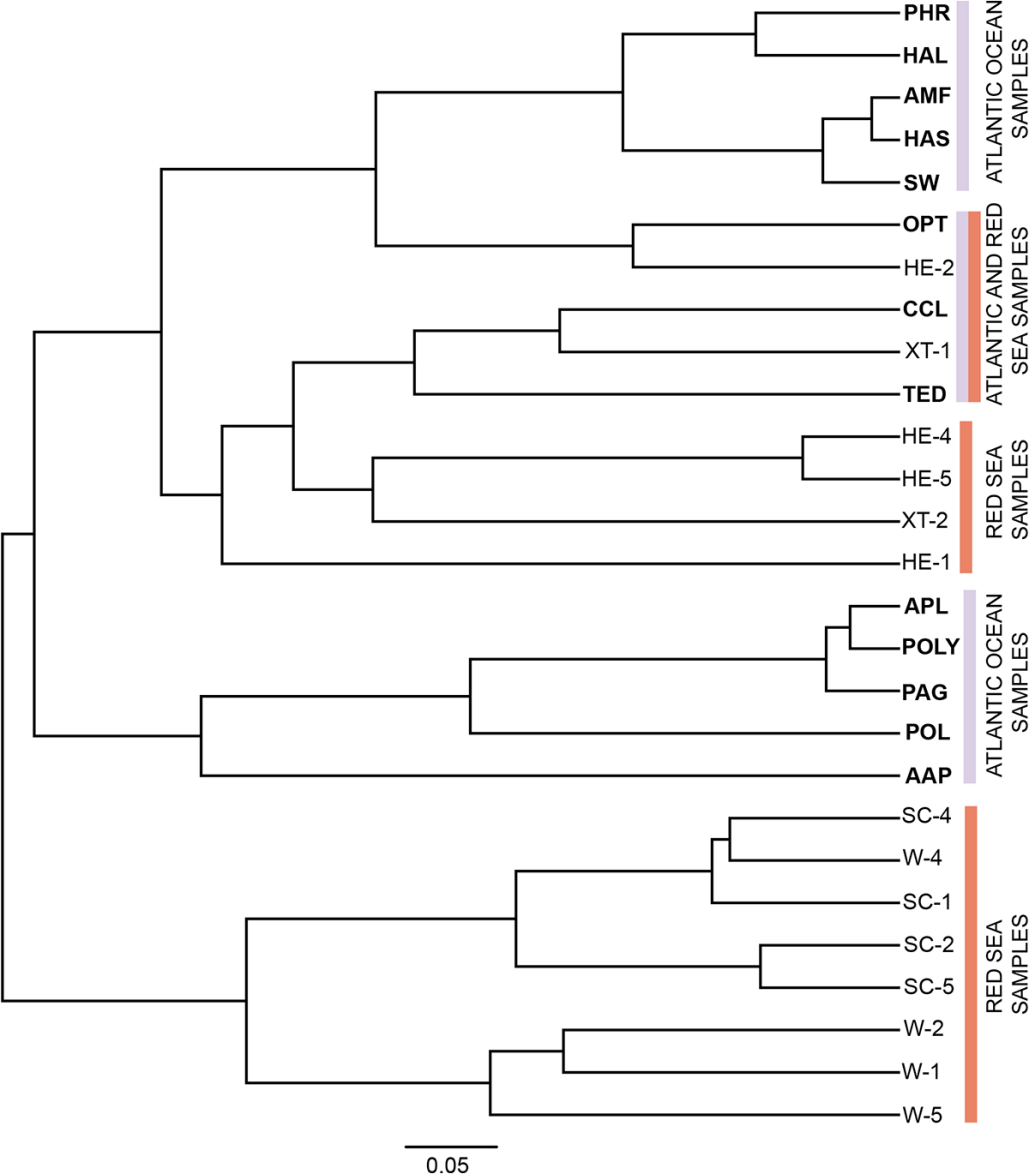
UPGMA cluster analysis of Crenarchaeotal communities associated with the Atlantic Ocean and the Red Sea samples. The bold letters indicate the samples from the current study. Detailed information about sample codes is provided in Table 1 (this study). Sequence data from the Red Sea samples were obtained from previous study [8].

**Fig 5:**
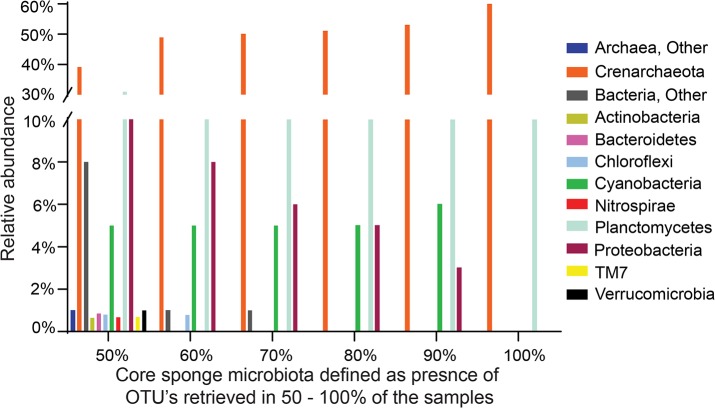
Core bacterial communities assigned to the sponge samples. The x-axis represents the core microbiata assignment defined as the distributions of the OTUs that are present in 50–100% of the sponge samples studied.

The non-metric multidimensional scaling (NMDS) plot clearly discriminated the bacterial communities among sample sources (represented by the lower value of stress 0.05891) (Fig 3). Microbial community differentiation using phylogenetic information (p-test) showed significant distinction (*p* < 0.01) of the microbes associated with the sponges and seawater (S6 Fig). In addition, we detected a bacterial community difference among three intrageneric sponge hosts (*Polymastia* sp.) studied (S7 Fig). The UPGMA cluster analysis of *Crenarchaeota* obtained here (Atlantic Ocean) and *Crenarchaeota* from the Red Sea sponges suggested the similarity of archaeal communities, but only between two Red Sea sponge species- *Hyrtios erectus* (HE-2) and *Xestospongia testudinaria* (XT-1), and three Atlantic Ocean sponge species- *Ophlitaspongia papilla* (OPT), *Tedania pillarriosae* (TED) and *C*. *celata* (CCL). Most of the sponges from both oceans were grouped separately indicating distinct *Crenarchaeota* communities (Fig 4). This pattern is further confirmed by nonparametric ADONIS (*p* = 0.0009, *R* ^*2*^ = 0.2) and ANOSIM (*p* = 0.001, *R* = 0.4206) analyses, revealing significant difference in the archaea communities from different oceans (S2 Table). We analyzed the distribution of core microbes among the sponge hosts, which are present in at least 50–100% of the samples. At the phylum level, both *Crenarchaeota* and *Planctomycetes* were found in all the sponge samples irrespective of the different core bacterial ranges defined (Fig 5).

**Fig 6:**
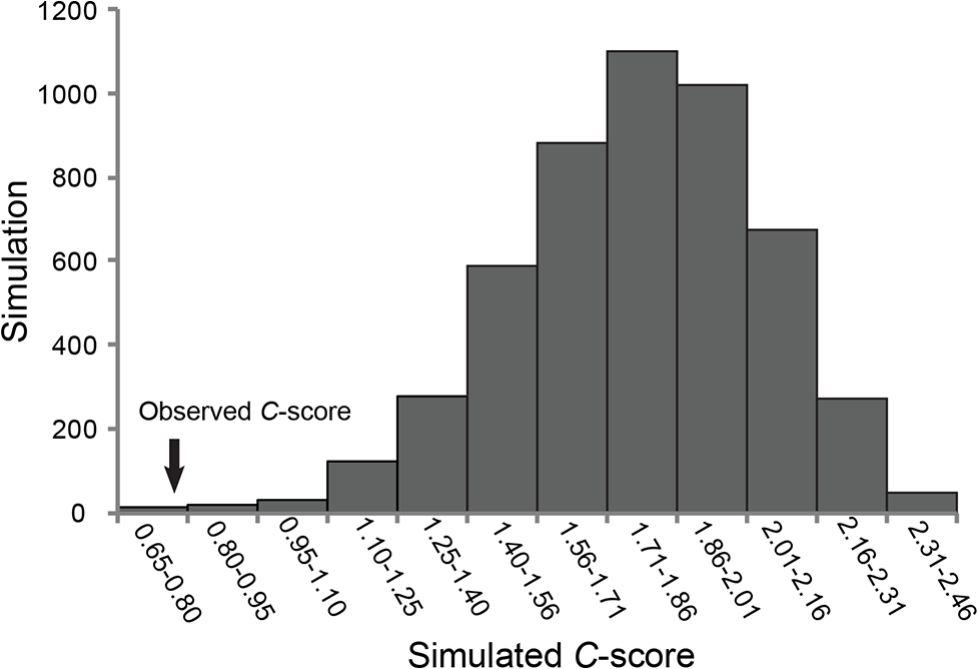
Histogram representation of the co-occurrence analysis. The observed C-score from the real data set (sponge associated microbes) is represented by an arrow on to the simulated C-scores.

The co-occurrence analysis did not support the non-random microbial distribution hypothesis among the sponge samples analyzed. At the phylum level, the observed C-score (0.74242) was significantly lower than the simulated C-score (1.78472; *p* < 0.001), suggesting a random microbial distribution pattern (Fig 6). Further analysis at the species level also supported the random distribution (null hypothesis) of sponge-associated bacteria (observed C-score = 2.885 and simulated C-score = 5.172; *p* < 0.001).

## Discussion

### Microbial consortium from sponges

Microbial assemblages in wide oceans are being documented both from open water (free-living bacteria) and in association with marine animals. Recently, sponges have been a major focus of study due to their microbial abundance, ecological role and biotechnological significance [32]. Here, we investigated the sponge-associated microbial assemblage from 12 different co-occurring intertidal marine sponges sampled from the Atlantic coast of Portugal and compared it with the surrounding ambient seawater from the sampling location. Tag pyrosequencing revealed altogether 26 different bacterial and archaeal phyla on the sponge communities (Fig 2) suggesting a complex microbial consortium. *Proteobacteria* formed the most abundant group of bacteria, distributed among all five major classes of *Alphaproteobacteria*, *Betaproteobacteria*, *Gammaproteobacteria*, *Deltaproteobacteria* and *Epsilonproteobacteria*. This bacterial group was very prominent in almost every sponges studied previously, irrespective of the habitat [32], and predominated during isolation techniques [54]. At the class level classification, the *Alphaproteobacteria* prevailed among all sponge samples followed by *Gammaproteobacteria* (S4 Fig). The second most diverse microbial group, *Crenarchaeota*, predominated both in seawater and sponge species. Other studies showed increasing evidence for the ubiquitous and abundant nature of archaeal communities [55,56]; which were previously thought to be present only in extreme conditions [57,58]. For instance, Crenarchaeotal microbes have been recovered from the open surface waters of temperate and polar seas [59,60], and other invertebrates beside sponges [61]. Interestingly, archaea are being found in association with marine sponges from diverse locations, such as Korea [62], Brazil [63] Antarctica [64] and the Red Sea [8]. We reported here for the first time the association of archaea, particularly the diverse *Crenarchaeota*, among 12 different intertidal marine sponge species from the European Atlantic coast (S5A Fig). High abundance of *Crenarchaeota* has previously been reported in the North Atlantic Ocean, playing an important role in the oxidation of ammonia to nitrite, indicated by the dominance of archaeal ammonia monooxygenase (amoA) genes [65]. The majority of archaeal reads (20.2%; S5B Fig) grouped in the genus *Nitrosopumilus* and were mostly found in the sponge *A*. *papillata* and less represented in *C*. *celata*. Overall, our study showed the presence of archaea among the intertidal sponges sampled from the Atlantic coast.

The high abundance of *Crenarchaeota* found in the Atlantic Ocean (this study) and the Red Sea sponges [8] prompted us to further investigate a possible similarity between archaeal communities from geographically separated sponges. A few samples from both oceans grouped differently indicating a clear distinction of multiple *Crenarchaeota* (Fig 4) microbial communities. The clustering of sponge samples from both the Red Sea (HE-1 and XT-1) and the Atlantic Ocean (OPT, CCL and TED) indicates the presence of similar microbial members of the archaeal lineage. However, the detection of *Crenarchaeota* may also suggest the generalist nature of these microbes. It is noteworthy that the retrieval of Crenarchaeotal group in this study could be due to their abundance and cosmopolitan distribution in the world’s oceans [55,59].

*Planctomycetes* and *Cyanobacteria* were also detected frequently in the sponge samples recovered from the intertidal region. Various studies have shown the association of photosynthetic bacteria and their benefits to sponges [15] inhabiting both in tropical [24] and temperate conditions [4,66,67]. It is evident that the majority of the sponges studied here (10 out of 12) harbored cyanobacteria (1.9%, including chloroplast; Fig 2). Lower level taxonomic classification revealed the presence of *Prochlorococcus*, a representative symbiotic cyanobacterial genera reported to colonize the widely studied sponges [68]. It is also possible that the detected cyanobacteria in the sponges sampled could be simply filtered food-particles. Since the intertidal sponges we studied are recurrently exposed to light during low tide, the prominence of photosymbionts might support the previous hypothesis of the cyanobacterial role in supplementing the sponges with required energy and protecting them from UV radiation during environmental exposure [24]. Moreover, we detected Candidatus *Entotheonella* from the sponges *Aplysilla rosea*, *Polymastia* sp. and *Aaptos papillata* in very low abundance (< 0.1%). *Entotheonella* sp. was previously identified in the marine sponge *Theonella swinhoei* [69] and *Discodermia* sp. [70,71].

Our data also revealed the ‘rare’ phyla *Chlamydiae* and WS3 at very low abundance (< 0.1%). Representatives of uncultivated bacterial members formed the ‘candidate divisions’ [72] that have been frequently reported from sponges [7–9] and other environments. Candidate bacteria of division TM7 previously reported to transmit vertically in the sponge *Xestospongia muta* [73] was also recovered from 10 sponge species and seawater sample in our study. However, retrieval of TM7 bacterial group might indicate the possible horizontal transmission of these microbes. Electron microscopic examination [74–76] and fluorescence *in situ* hybridization (FISH) [73,77] of the adult as well as the sponge larvae could explain the mode of microbial transmission in the current sponge samples. Moreover, we detected the presence of two new sponge-associated uncultivated bacterial groups: the candidate phyla NKB19 and ZB2, which highlights the usefulness of deep sequencing in our study.

### Lack of non-random association among sponge microbiota

The majority of microbes co-exist in different mode of association within the ecosystem and non-random patterns of interaction are known to exist across all domains of life [78]. These associations play an important role in the structuring of the microbial communities [79] through microbe-microbe and microbe-metazoan interactions. The cataloging of such ecological patterns is important for understanding the ecosystem dynamics and the evolutionary ecology of individual organisms [80]. Considering the exceptionally high microbial diversity in sponges, it is always tempting to conclude a non-random pattern of microbial association, where microbial taxa are segregated and exhibit close relationships. However, our results do not support a non-random microbial association in the sponges studied (i.e., significantly less observed C-score; Fig 6), which suggests that other factors may influence the structuring of the microbial assemblage in sponges. Co-occurrence analysis considered the bacterial communities among varying gradients like different human genotypes [81] and different soil types [35], validating the non-random association. Even though we tested the co-occurrence hypothesis among the sponges sampled from similar external gradients (temperature, salinity and pH), there might be oscillating internal biotic factors among the sponge species influencing the microbial community structuring. Abiotic factors, mainly temperature, can substantially influence the sponge holobionts due to the removal of symbionts and the immediate introduction of opportunistic bacteria [82]. Although there are widespread competition between microbes for resources, its detection in natural environment is not trivial [80]. Our current dataset is not exhaustive to explain the co-occurrence pattern in sponge microbes, and further studies should follow with wide sampling from different ecosystems.

### Sponge-associated microbes are not restricted to its host

It is noteworthy that nearly 73% of the microbial groups retrieved in this study were represented both in sponges and seawater samples. A decade ago sponge microbiology proposed two definitions—‘sponge-specific’ and ‘sponge-species-specific’ [31] to describe the particular nature of the microbial association with sponges which was further validated later [83]. In our study we have not defined the “sponge-specific” or “sponge-species-specific” microbial communities due to the recurrent presence of similar microbes among the sponge species and ambient seawater. It is clear from our dataset that *Chlamydiae*, *Nitrospirae*, and the two candidate phyla SBR1093 and TM6 were the only microbial groups found exclusively in association with the sponges. A recent comprehensive study [84] substantiated the widespread (but rare) existence of microbes in diverse marine environments previously thought to be present only in sponges. The presence of such microbes suggests its ability to survive outside the sponge host, which may serve as a ‘seed bank’ for the colonization of sponges [7]. Further rigorous sampling and deep-sequencing could provide valuable knowledge to understand the nature of the microbial specificity among these sponges.

## Conclusion

Here, we provide the first report on the sponge-associated microbial communities in intertidal Atlantic sponges. 16S rRNA tag pyrosequencing exposed a diverse and complex nature of sponge-associated microbes among 12 different co-occurring intertidal sponges (class Demospongiae) from the Atlantic coast of Portugal. OTU definition of microbial communities at 97% similarity threshold revealed altogether 26 different microbial groups, including new bacterial groups (candidate phyla NKB19 and ZB2) not detected previously in sponges. Comparison of the sponge-associated archaeal communities suggests similarity of *Crenarchaeota* between geographically isolated Atlantic Ocean and distant Red Sea sponges. However, the observation of sponge-associated microbial communities in ambient seawater suggests that they can be widespread in marine environments, existing outside of the sponge body either in an active or inactive state. The flexibility of sponge-associated microbes to either flourish in seawater or in association with sponge host in later stages of life may contribute to the lack of co-occurrence patterns. Further detailed evaluation using different gradients at spatial and temporal scale would be insightful to clarify the co-occurrence pattern among sponges.

## Supporting Information

S1 Fig Total number of OTUs represented at different similarity thresholds. Pyrosequencing reads are grouped at various sequence similarity revealing lesser number of OTUs with less sequence similarities.
(PDF)

S2 Fig Graph representing total number of sequencing reads and OTUs recovered from the samples. A. Number of reads retrieved from each sample after quality filtering. B. Number of OTUs represented in each sample. Detailed information about sample codes is provided in Table 1.
(PDF)

S3 Fig Rank abundance curve showing diversity of bacterial communities associated with sponge species and seawater (SW). Detailed information about sample codes is provided in Table 1.
(PDF)

S4 Fig Relative abundance of *Proteobacteria* among the sample sources. Detailed information about sample codes is provided in Table 1.
(PDF)

S5 Fig Relative abundance of archaea bacteria from the samples. (A) Relative abundance of the phyla *Crenarchaeota* and *Euryarchaeota*. (B) Abundance of archaea bacteria at lower taxonomic level. Sample code details are given in Table 1.
(PDF)

S6 Fig Pairwise comparison of the microbial communities amongst the different sponge species and seawater (SW). Heat map representing the *p*-values obtained from the p-test [86] performed for each pair of samples. Comparison of differentiation among microbes in all possible pair of samples was calculated using phylogenetic information. Similarities between microbial communities are estimated as the number of parsimony changes and the *p*-values (< 0.01) explains the probability that the assigned sample pairs are dissimilar. Colors are coded in the cell according to significance values. Sample code details are given in Table 1.
(PDF)

S7 Fig Heat map showing pairwise dissimilarity between intergeneric sponge-associated bacterial communities. Beta-diversity metrics, pearson distance was used to compute the differences among microbes in three different sponge species, *Polymastia agglutinans* (PAG), *Polymastia penicillus* (POLY) and *Polymastia* sp. (POL). The resulting distance matrices were plotted as a heat map, with increasing grades of blue color representing greater dissimilarity.
(PDF)

S1 Table List of samples and respective barcodes. Multiplex Identifiers (MID) were attached with 16S rRNA primer for amplifying each sample and used like a barcode to identify amplicons or samples during pyrosequencing.
(DOCX)

S2 Table Statistical test of sample groupings. Statistical significance of grouping of the Atlantic Ocean (this study) and the Red Sea samples analyzed with compare_catergories.py function in QIIME using Bray-Curtis distance matrix derived from the *Crenarchaeota* communities. The symbol ‘*’ represents significant p-values obtained from the test.
(DOCX)
