# Supplementary figures and images for: Pyrosequencing Characterization of the Microbiota from Atlantic Intertidal Marine Sponges Reveals High Microbial Diversity and the Lack of Co-Occurrence Patterns
Source: PLoS One. 2015 May 20;10(5):e0127455. doi: 10.1371/journal.pone.0127455 (PMC4439068; doi:10.1371/journal.pone.0127455)

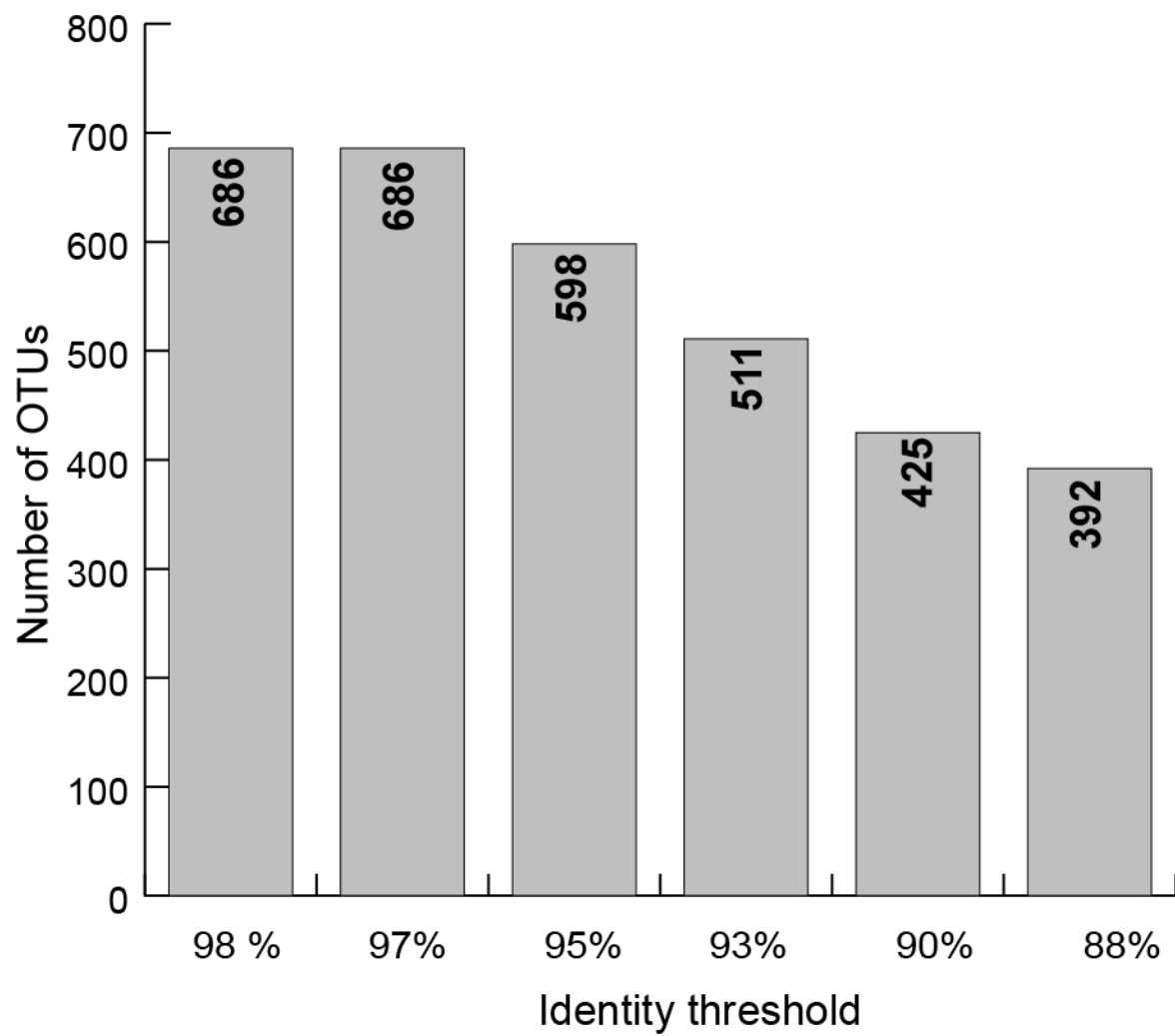

Supplement: S1 Fig — Pyrosequencing reads are grouped at various sequence similarity revealing lesser number of OTUs with less sequence similarities. (PDF) [file pone.0127455.s001.pdf]

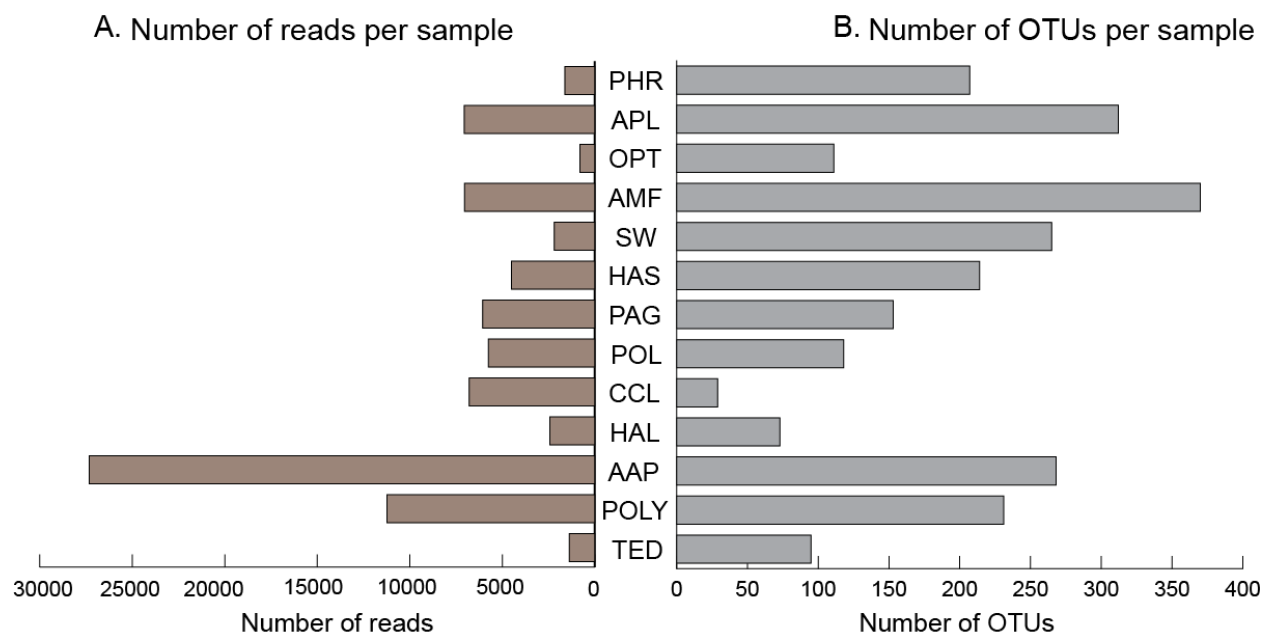

Supplement: S2 Fig — A. Number of reads retrieved from each sample after quality filtering. B. Number of OTUs represented in each sample. Detailed information about sample codes is provided in Table 1. (PDF) [file pone.0127455.s002.pdf]

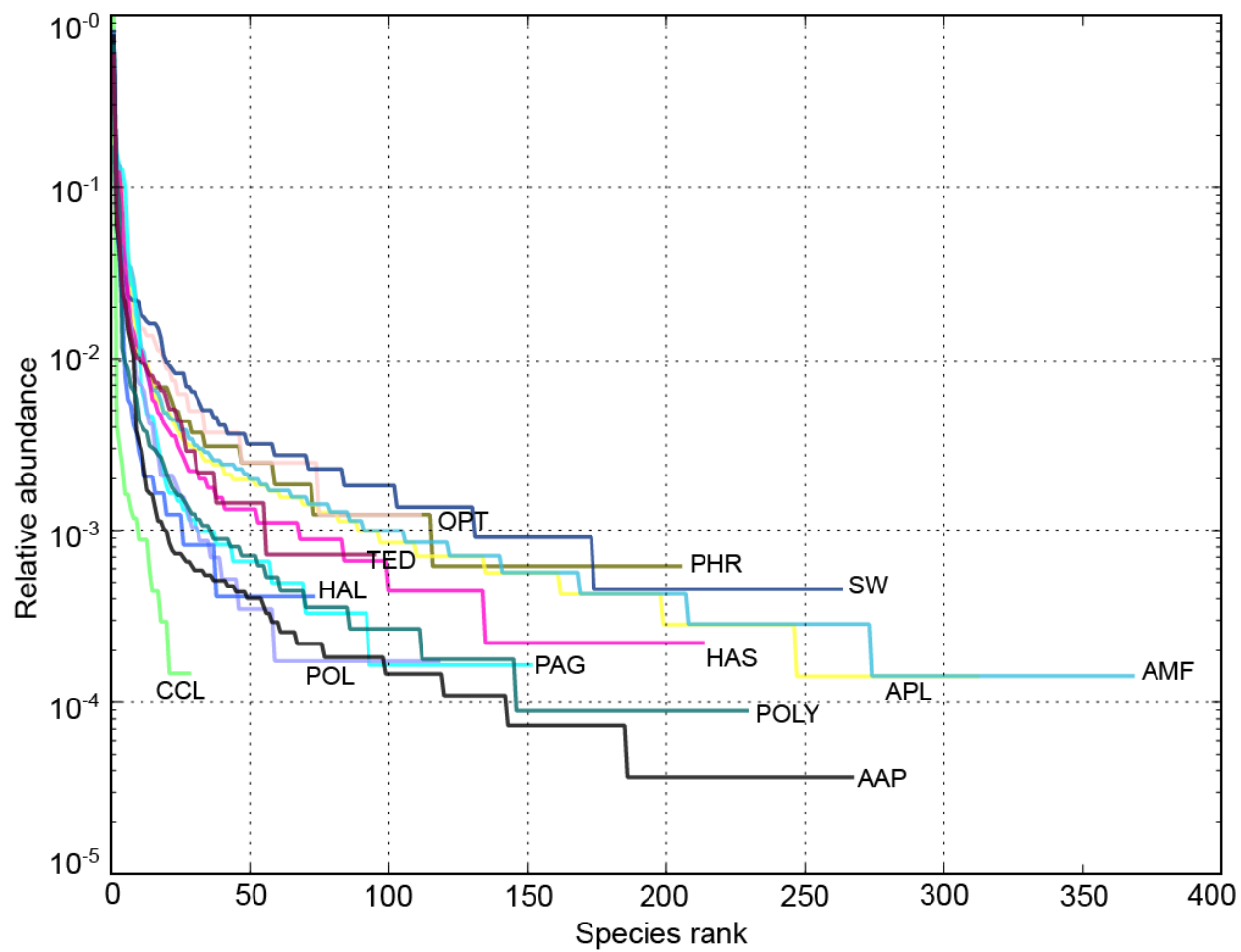

Supplement: S3 Fig — Detailed information about sample codes is provided in Table 1. (PDF) [file pone.0127455.s003.pdf]

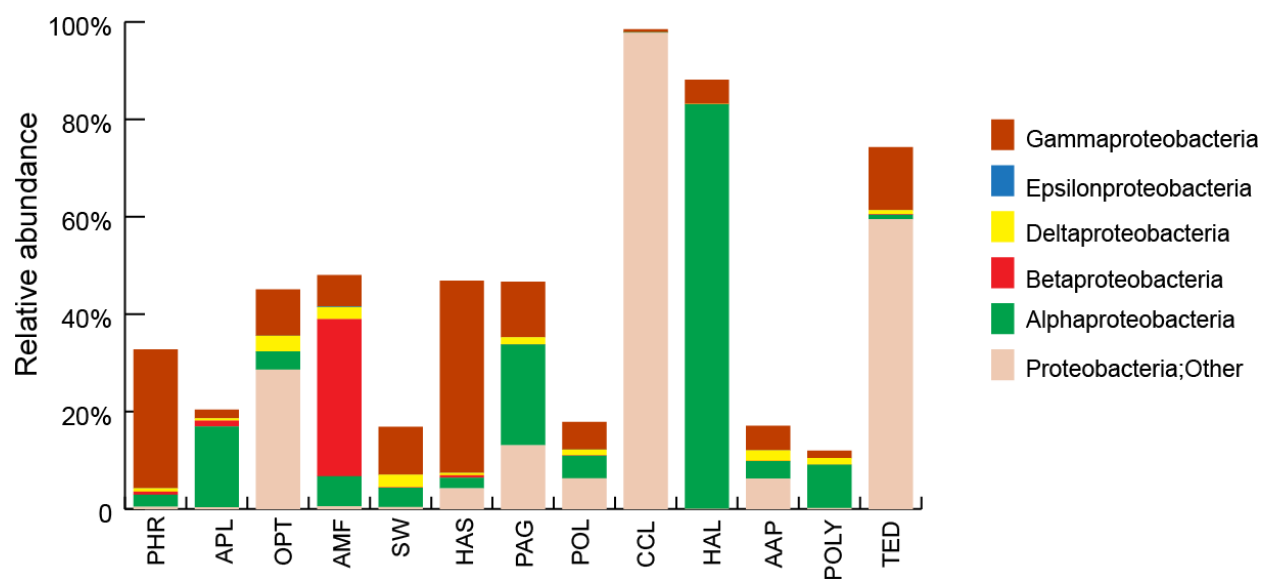

Supplement: S4 Fig — Detailed information about sample codes is provided in Table 1. (PDF) [file pone.0127455.s004.pdf]

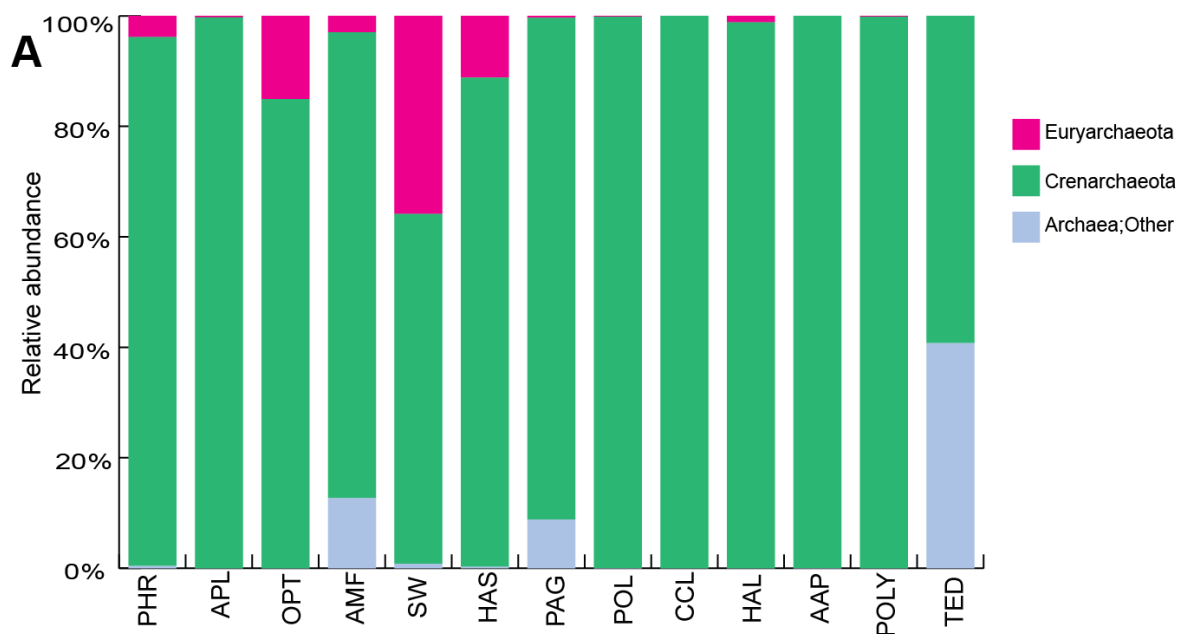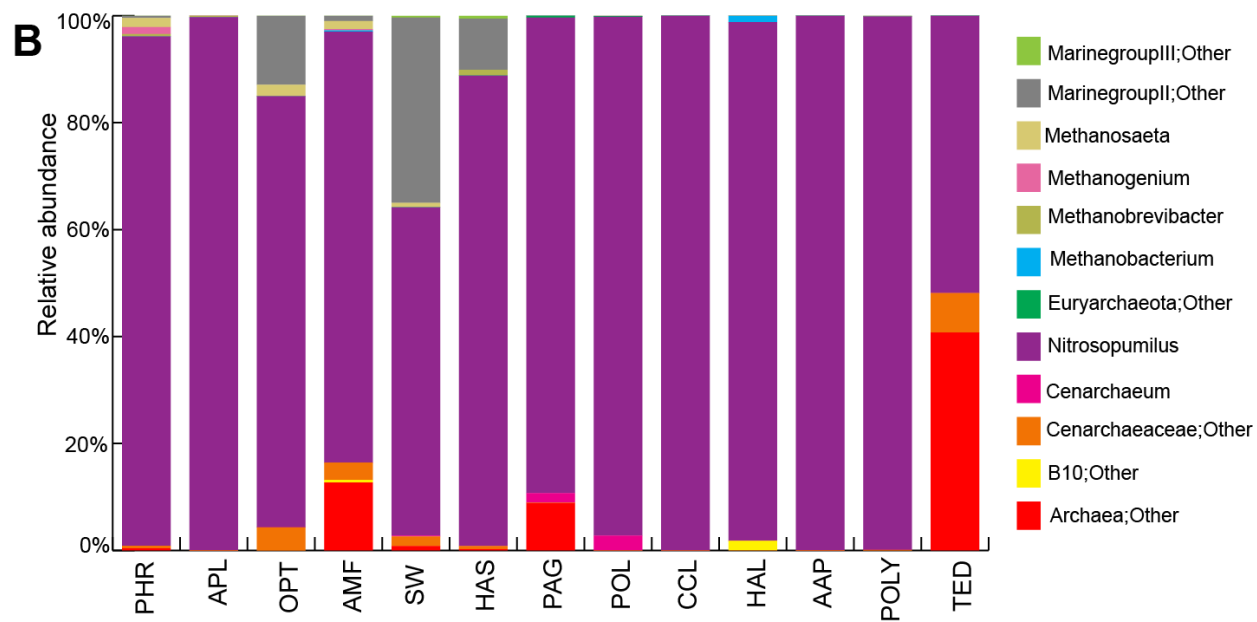

Supplement: S5 Fig — (A) Relative abundance of the phyla Crenarchaeota and Euryarchaeota. (B) Abundance of archaea bacteria at lower taxonomic level. Sample code details are given in Table 1. (PDF) [file pone.0127455.s005.pdf]

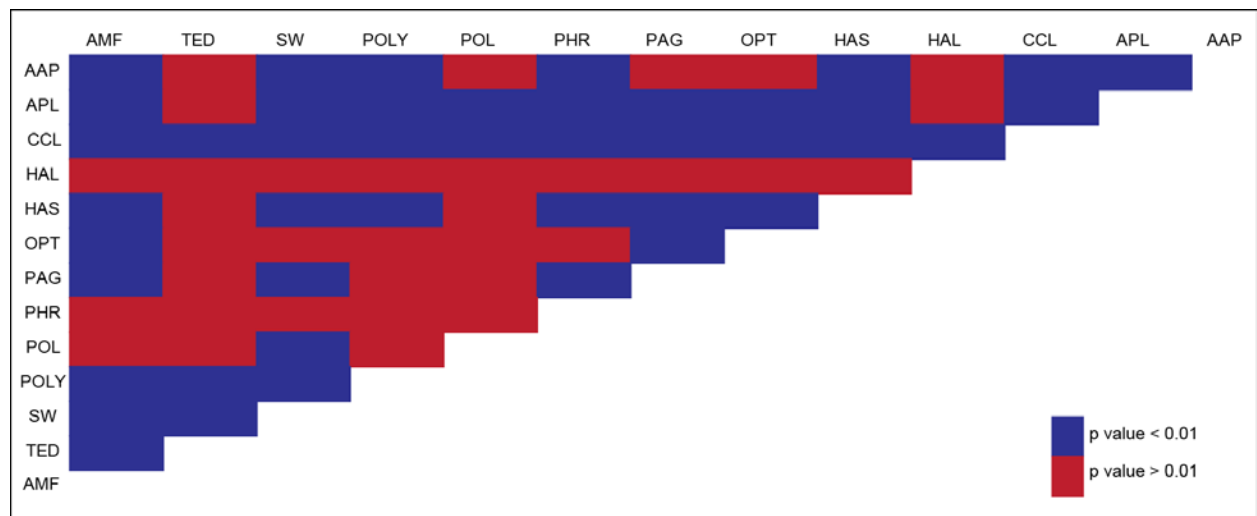

Supplement: S6 Fig — Heat map representing the p-values obtained from the p-test [86] performed for each pair of samples. Comparison of differentiation among microbes in all possible pair of samples was calculated using phylogenetic information. Similarities between microbial communities are estimated as the number of parsimony changes and the p-values (< 0.01) explains the probability that the assigned sample pairs are dissimilar. Colors are coded in the cell according to significance values. Sample code details are given in Table 1. (PDF) [file pone.0127455.s006.pdf]

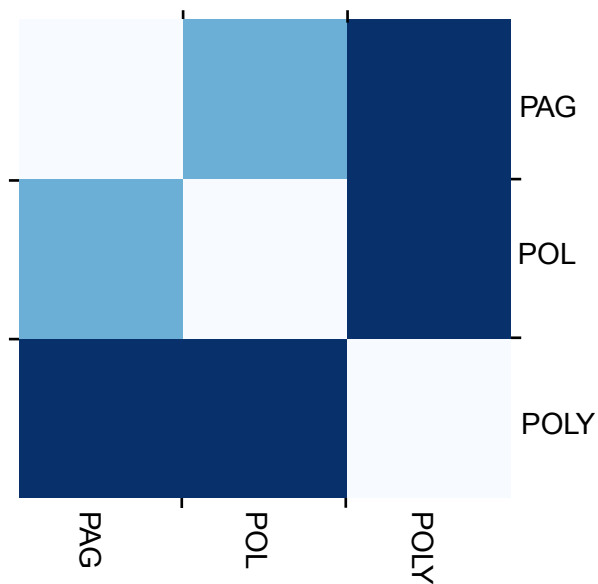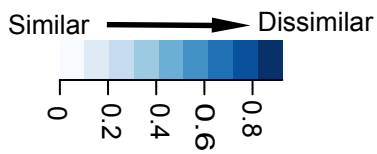

Supplement: S7 Fig — Beta-diversity metrics, pearson distance was used to compute the differences among microbes in three different sponge species, Polymastia agglutinans (PAG), Polymastia penicillus (POLY) and Polymastia sp. (POL). The resulting distance matrices were plotted as a heat map, with increasing grades of blue color representing greater dissimilarity. (PDF) [file pone.0127455.s007.pdf]
