# Supplementary material for: Pyrosequencing Characterization of the Microbiota from Atlantic Intertidal Marine Sponges Reveals High Microbial Diversity and the Lack of Co-Occurrence Patterns
Source: PLoS One. 2015 May 20;10(5):e0127455. doi: 10.1371/journal.pone.0127455 (PMC4439068; doi:10.1371/journal.pone.0127455)
Supplement: S1 Table — Multiplex Identifiers (MID) were attached with 16S rRNA primer for amplifying each sample and used like a barcode to identify amplicons or samples during pyrosequencing. (DOCX) [file pone.0127455.s008.docx]

**S1 Table. List of samples and respective barcodes.**

| List of samples | Sample code | MID |
| --- | --- | --- |
| *Amphilectus* *fucorum* | AMF | ATACGACGTA |
| *Aplysilla rosea* | APL | CGAGAGATAC |
| *Aaptos papillata* | AAP | AGCACTGTAG |
| *Cliona celata* | CCL | CATAGTAGTG |
| *Haliclona simulans* | HAS | TCTCTATGCG |
| *Halichondria panicea* | HAL | ATATCGCGAG |
| *Ophlitaspongia papilla* | OPT | ATCAGACACG |
| *Polymastia agglutinans* | PAG | ACGAGTGCGT |
| *Polymastia* *penicillus* | POLY | ACGCTCGACA |
| *Polymastia* sp. | POL | AGACGCACTC |
| *Phorbas plumosus* | PHR | CGTGTCTCTA |
| *Tedania pillarriosae* | TED | CTCGCGTGTC |
| Seawater | SW | TCACGTACTA |
