# Supplementary material for: Pyrosequencing Characterization of the Microbiota from Atlantic Intertidal Marine Sponges Reveals High Microbial Diversity and the Lack of Co-Occurrence Patterns
Source: PLoS One. 2015 May 20;10(5):e0127455. doi: 10.1371/journal.pone.0127455 (PMC4439068; doi:10.1371/journal.pone.0127455)
Supplement: S2 Table — Statistical significance of grouping of the Atlantic Ocean (this study) and the Red Sea samples analyzed with compare_catergories.py function in QIIME using Bray-Curtis distance matrix derived from the Crenarchaeota communities. The symbol ‘*’ represents significant p-values obtained from the test. (DOCX) [file pone.0127455.s009.docx]

| **(a) ANOSIM** | R statistics | p-value | Number of permutations |
| --- | --- | --- | --- |
|  | 0.4206 | 0.001* | 1000 |

**S2 Table. Statistical test of sample groupings.**

| **(b) ADONIS** | Df | SumsOfSeqs | MeanOfSeqs | F.Model | R2 | Pr(>F) |
| --- | --- | --- | --- | --- | --- | --- |
| Data | 1 | 2.0284 | 2.02842 | 6.3526 | 0.20262 | 0.000999* |
| Residuals | 25 | 7.9827 | 0.31931 |  | 0.79738 |  |
| Total | 26 | 10.0111 |  |  | 1.00000 |  |
